# An interpretable deep learning framework for predicting liver metastases in postoperative colorectal cancer patients using natural language processing and clinical data integration

**DOI:** 10.1002/cam4.6523

**Published:** 2023-09-11

**Authors:** Jia Li, Xinghao Wang, Linkun Cai, Jing Sun, Zhenghan Yang, Wenjuan Liu, Zhenchang Wang, Han Lv

**Affiliations:** ^1^ Department of Radiology Beijing Friendship Hospital, Capital Medical University Beijing People's Republic of China; ^2^ School of Biological Science and Medical Engineering Beihang University Beijing People's Republic of China; ^3^ Department of Radiology, Aerospace Center Hospital Beijing People's Republic of China

**Keywords:** artificial intelligence, bidirectional encoding representation of transformer, electronic health records, interpretable deep learning, natural language processing

## Abstract

**Background:**

The significance of liver metastasis (LM) in increasing the risk of death for postoperative colorectal cancer (CRC) patients necessitates innovative approaches to predict LM.

**Aim:**

Our study presents a novel and significant contribution by developing an interpretable fusion model that effectively integrates both free‐text medical record data and structured laboratory data to predict LM in postoperative CRC patients.

**Methods:**

We used a robust dataset of 1463 patients and leveraged state‐of‐the‐art natural language processing (NLP) and machine learning techniques to construct a two‐layer fusion framework that demonstrates superior predictive performance compared to single modal models. Our innovative two‐tier algorithm fuses the results from different data modalities, achieving balanced prediction results on test data and significantly enhancing the predictive ability of the model. To increase interpretability, we employed Shapley additive explanations to elucidate the contributions of free‐text clinical data and structured clinical data to the final model. Furthermore, we translated our findings into practical clinical applications by creating a novel NLP score‐based nomogram using the top 13 valid predictors identified in our study.

**Results:**

The proposed fusion models demonstrated superior predictive performance with an accuracy of 80.8%, precision of 80.3%, recall of 80.5%, and an F1 score of 80.8% in predicting LMs.

**Conclusion:**

This fusion model represents a notable advancement in predicting LMs for postoperative CRC patients, offering the potential to enhance patient outcomes and support clinical decision‐making.

## 1 INTRODUCTION

Colorectal cancer (CRC)^1^ is the third most common malignancy worldwide (10.0%) and the second most common cause of cancer‐related deaths (9.4%). With the ongoing research on molecular mechanisms of cancer and the joint development of various omics studies, an increasing number of treatment options are now available for local lesions and advanced diseases, thereby improving individualized diagnosis, treatment, and precision medicine.^2^ Current treatments for CRC include endoscopic and surgical local excision, downstaging preoperative radiotherapy and systemic therapy, extensive surgery for local and metastatic disease, local ablation of metastases, palliative chemotherapy, targeted therapy, and immunotherapy. These treatments, alone or in combination, significantly improve the survival of CRC patients. The liver is the most common site of postsurgery metastasis, involved in 25%–50% of CRC patients during the follow‐up period.^3^ Liver metastatic lesions detected in the early stage can be removed by surgery, resulting in a better overall prognosis. However, only 25% of the patients are suitable for first‐line therapy at the time of CRC liver metastasis (LM) diagnosis,^4^ owing to the rapid metastases. As a result, most patients receive second‐line chemotherapy as an alternative, associated with greater toxicity and a worse prognosis. Therefore, it has always been a challenge to predict LM in patients with CRC.

Radiological techniques are the most promising for the surveillance of LMs in CRC patients. Experts have developed standards for evaluating liver lesions, such as the Liver Reporting & Data System (LI‐RADS®). However, the frequency at which imaging tests should be performed to prevent postoperative recurrence has been controversial. Although recent studies have shown that 16%–26% of liver lesions are too small to be identified or excluded as benign lesions,^5^ invasive physical examinations, such as needle biopsies, are not recommended because their benefits may not outweigh the risk and cost to the patients. Moreover, repeated CT scanning may increase the risk of tumor mutation and progression, especially considering the aggressive nature of CRC metastasis.^6^ Thus, a valid analytical strategy for assessing and predicting LM in postoperative CRC patients, through which physicians can gain more confidence in determining whether a radiology examination should be scheduled for personalized surveillance of LM, can be attractive in clinical scenarios. In addition, such strategy would promote more efficient use of imaging techniques and improve the overall well‐being of CRC patients.

With the rapid development of artificial intelligence (AI) and big data, medical multimodal big‐data‐driven algorithms have achieved remarkable breakthroughs. Radiomics and pathomics^7^, ^8^, ^9^ have successfully predicted the prognosis and assessed the risk of metastasis in CRC patients.^10^ In a retrospective study by Li et al. on data from 766 patients undergoing LM resection, a neural network model was developed to predict the overall survival (OS) more accurately than the Cox regression model.^11^ More recently, Wang et al.^12^ reported a multiomics model by combining pathomics, radiomic features, immune scores, and clinical factors into a novel nomogram with outstanding performance in predicting OS (area under the curve [AUC] 0.860) and disease‐free survival (AUC 0.875). These breakthroughs inspire future research to develop more advanced AI techniques to improve the overall efficacy of CRC treatment.

Despite significant success in predicting LMs using the multiomic approach, unneglectable barriers hinder the clinical application of those models. For example, the data quality must meet the unified standard set by the model‐builder to ensure the consistency of model input, which is difficult to satisfy in real‐world clinical scenarios owing to the variation in data acquisition techniques and a lack of comprehensive quality assessment method.^13^ Moreover, many patients may not choose to visit the same hospital during follow‐up, which indicates that multiomic data may not be available to the physicians in terms of original electronic profiles during subsequent visits, mainly owing to legal obstacles associated with transferring between electronic health record (EHR) systems across different hospitals.^14^ In this regard, the clinical history can offer important evidence such as the duration of the disease, treatment records, changes in symptoms, and comprehensive summarization made by the previous physician, which provides a full review of the patients in unstructured texts. It is highly attractive to develop novel approaches to use these informatic data in AI models to prompt computer‐aided diagnosis.

Natural language processing (NLP) is an essential branch of AI technology that aims to convert natural language into a computable digital form to achieve text‐level understanding and calculation. In the medical domain, the mainstream of NLP focuses on extracting clinically meaningful entities or classifying subgroups using EHRs, radiology reports, or drug instructions. Moreover, studies utilizing deep learning from free text to predict patient outcomes deserve further attention. More recently, Causa Andrieu et al.^15^ developed an NLP‐based radiology report analysis model to identify clinically meaningful CRC metastatic phenotypes and demonstrated a correlation between the phenotypes and overall clinical survival. Our previous study established a domain‐specific transfer learning pipeline to identify patients with clinically meaningful pathogenesis related to tinnitus.^16^ However, the integration of multimodal data, such as free text, genomics, and radiomics, and structured data has always been a critical challenge in modeling.

This study aimed to effectively quantify the risk of LM in CRC patients using EHRs and laboratory data by constructing a novel fusion framework. The highlights of this study are listed as follows:

### 1.1 Highlights of this study

1. A two‐tier fusion‐based framework is proposed to predict LMs in CRC patients. A total of 18 structured clinical factors including age, gender, the most recent laboratory tests associated with liver function, and cancer metastasis, in addition to clinical history, intraoperative findings, and pathology phenotypes from original medical record, have been manually extracted and numerized. Moreover, deep learning‐based textual features based on the most recent medical record have been modeled as free‐text representative features and included in the modeling.
2. We have established a novel NLP and clinical factors‐based nomogram for the practical application of our fusion model. As clinical texts are the most common and essential data collected during the follow‐up of CRC patients, this nomogram may have broader applications.
3. We evaluated the contribution of each data module to the prediction accuracy during the fusion process, thus improving the interpretability of this complex model.

## 2 MATERIALS AND METHODS

### 2.1 Study overview

**FIGURE 1:**
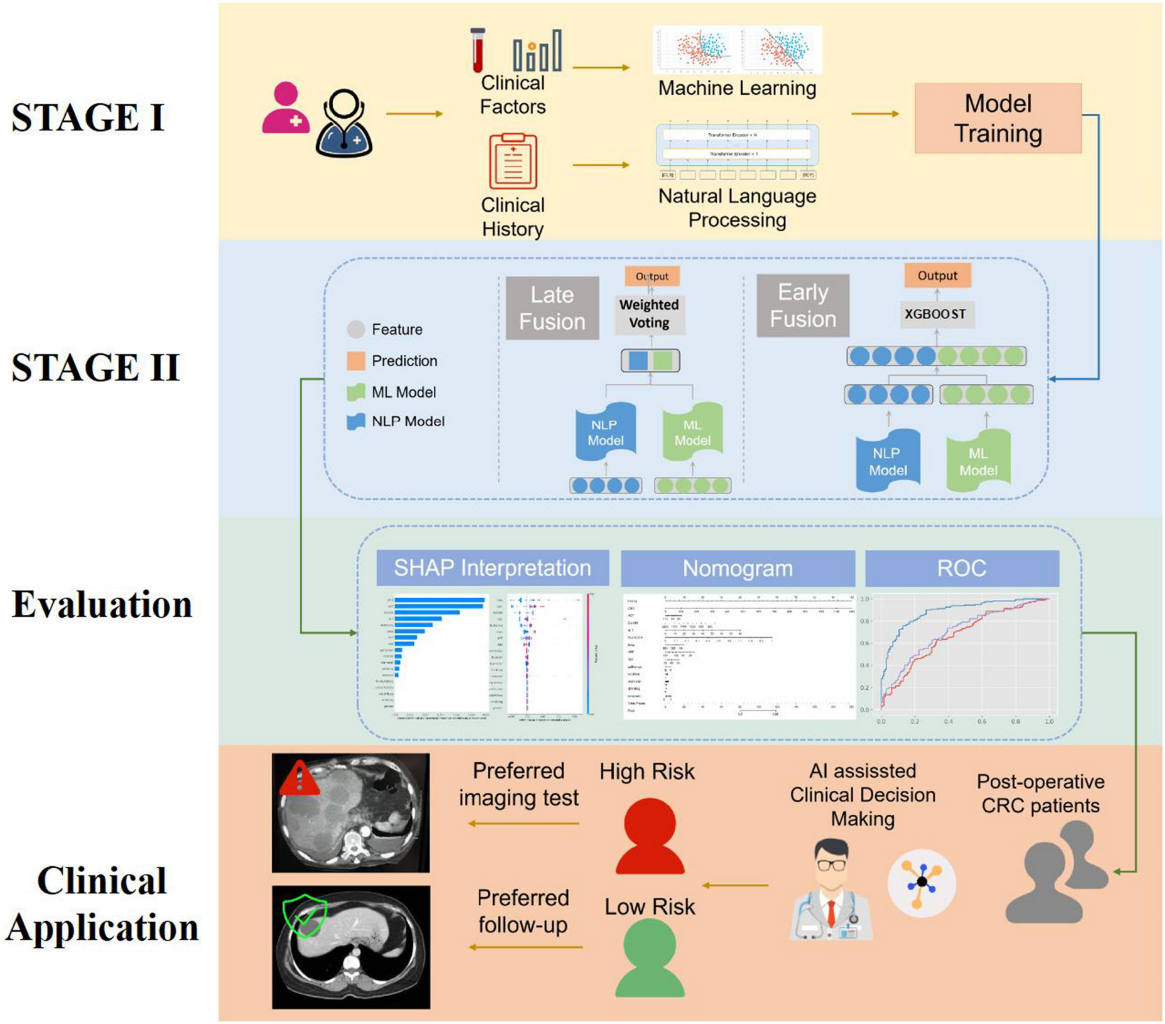
Study workflow of this study: in Stage I, machine learning (ML) and natural language processing (NLP) models were trained respectively; in Stage II, two fusion approaches were used to combine and integrate the prediction information of each model; then, ROC, Shapley additive explanations (SHAP), and nomograms were used as evaluation and explanation tools; finally, we propose the utilization of the proposed model by quantifying the liver metastasis (LM) risk of colorectal cancer (CRC) postoperative patients.

This study consisted of four parts. In Part 1, we built the machine learning (ML) and NLP models using structured clinical factors and free‐text medical history to evaluate their accuracy in predicting LMs. In Part 2, we used two advanced fusions, namely stacking and ensembling methods. Thus, a fusion learning framework was established to realize the joint prediction of LM by ML and NLP models. In the third part, the model performance was evaluated in terms of accuracy, precision, recall, F1, receiver operating characteristic (ROC) curve, AUC, and Shapley additive explanations (SHAP) values to improve the interpretability of the model. In the fourth part, we constructed a novel nomogram based on clinical factors and NLP scores to provide a valuable tool for clinical applications. Figure 1 presents the workflow of this study.

The study was conducted according to the Declaration of Helsinki. It was approved by the Beijing Friendship Hospital Ethics Committee, Capital Medical University (Research Application System number 2021‐P2‐144‐01), and “Ethical Review of Biomedical Research Involving People,” the Ministry of Public Health of China.

### 2.2 Data collection and label definition

We retrospectively collected EHR data from a tertiary hospital in Beijing, China, including the data of 1463 CRC patients admitted for surgery and followed‐up between 2019 and 2022. All authors discussed the inclusion and exclusion criteria. All definitions and details are listed in Table S1 in Data S1.

All structured clinical data were derived from the Hospital Information System (HIS), including general information, laboratory test results, surgical record findings, and pathology results. Clinical free‐text medical history was defined as admission history at the most recent follow‐up visit, and follow‐up time was defined as the time between the initial surgery and the most recent follow‐up visit. We used two independent sample *t*‐tests to analyze the differences between the groups.

**FIGURE 2:**
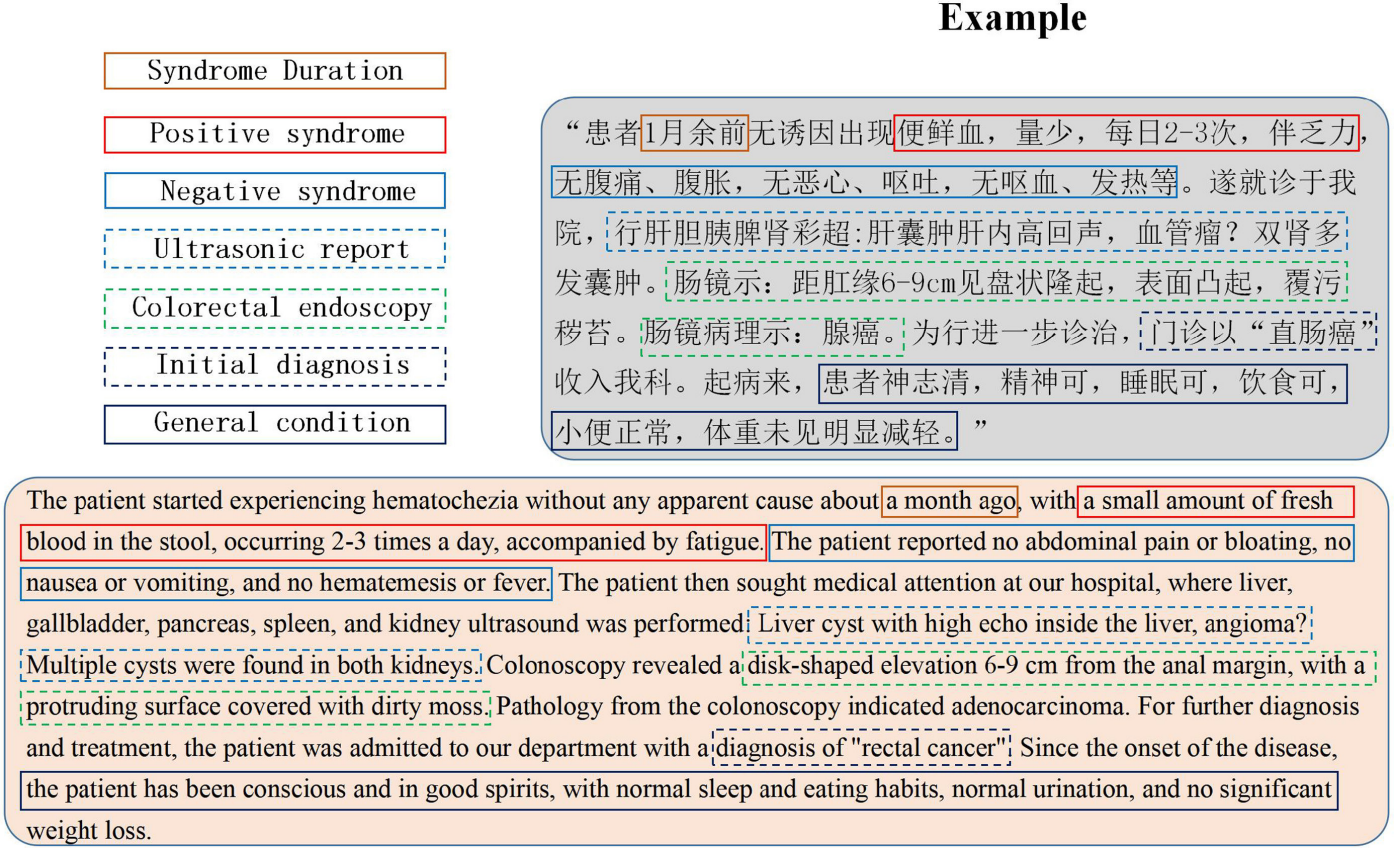
Example of an original clinical note in Chinese (upper right corner). Typically, each note provides six‐dimensional information, including positive symptoms, negative symptoms, laboratory results, imaging test results (e.g., ultrasound and colorectal endoscopy), the initial diagnosis, and a summary of the general condition. Below the original note, an English translation of the clinical note is provided.

The clinical notes used in this study were not annotated and acquired from the patients' most recent visits. These notes offer a comprehensive record of the patients' entire medical journey since the onset of the disease, encapsulating primary symptoms, duration, treatment process, and other pertinent information. An example is shown in Figure 2. Note that this contextual information was processed by the NLP model without additional labeling. A sample of clinical notes used in our study is provided in Data S2.

All patients underwent standard procedures for LM screening on admission: a CT scan of the upper abdomen with contrast or an MRI with contrast. The diagnostic criteria for LMs were determined using the LI‐RADS@ criteria defined by the American College of Radiology.^17^ Based on the imaging features, liver lesions are scored as LR‐1 (100% benign), LR‐2 (probably benign), LR‐3 (intermediate probability for HCC), LR‐4 (probably HCC), and LR‐5 (100% definite HCC). As per the diagnostic criteria of recent international large‐scale clinical trials,^18^ This study defined “No metastasis” as the following three conditions: no nodules detected, presence of LR‐1 lesions, or the presence of LR‐2 lesions. “Metastasis” was defined as detection of at least one LR‐3 to LR‐5 lesion. Table 1 compares the basic statistics between the two groups.

**TABLE 1:** Structured and semi‐structured data on 18 characteristics for 1463 colorectal cancer patients were included in this study.

		No metastasis group *n* = 854	Metastasis group *n* = 609	*p*‐Value
General information	Age (year)	67.18 ± 11.798	67.73 ± 11.661	0.378
	Sex			
	Male	502 (0.587)	397 (0.651)	0.013
	Female	352 (0.412)	212 (0.348)	
Laboratory information	AST (U/L)	17.63 ± 6.983	19.87 ± 10.933	< 0.001
	ALT (U/L)	13.91 ± 9.080	16.41 ± 13.592	< 0.001
	AFP (IU/mL)	3.04 ± 5.218	3.15 ± 6.049	0.705
	CEA (ng/mL)	11.07 ± 35.728	55.21 ± 177.338	<0.001
	CA199 (ku/L)	26.7 2 ± 78.864	114.55 ± 344.838	<0.001
Clinical history	Smoking			
	No	583 (68.27%)	379 (62.23%)	0.016
	Yes	271 (31.73%)	230 (37.77%)	
	Drinking			
	No	620 (72.60%)	419 (68.80%)	0.115
	Yes	234 (27.40%)	190 (31.20%)	
	Weight loss			
	No	544 (63.70%)	366 (60.10%)	0.162
	Yes	310 (36.30%)	243 (39.90%)	
	Cancer History			
	No	750 (87.82%)	538 (88.34%)	0.763
	Yes	104 (12.18%)	71 (11.66%)	
	Family History			
	No	822 (96.25%)	590 (96.88%)	0.519
	Yes	32 (3.75%)	19 (3.12%)	
Intraoperative findings	Diameter (cm)	4.78 ± 2.163	5.04 ± 2.205	0.023
	Invasion Range (%)	0.74 ± 0.263	0.76 ± 0.254	0.075
	Location			
	Lower rectum	76 (8.90%)	44 (7.22%)	0.079
	Middle rectum	139 (16.28%)	90 (14.78%)	
	Upper rectum	149 (17.45%)	112 (18.39%)	
	Sigmoid colon	263 (30.80%)	184 (30.21%)	
	Descending colon	61 (7.14%)	40 (6.57%)	
	Transverse colon	33 (3.86%)	22 (3.61%)	
	Ascending colon	133 (15.57%)	117 (19.21%)	
Pathological information	Pathology Type			
	Uplift	244 (28.57%)	185 (30.38%)	0.428
	Ulcer	603 (70.61%)	420 (68.97%)	
	Infiltration	7 (0.82%)	4 (0.66%)	
	Differentiation			
	Poor	110 (12.88%)	90 (14.78%)	0.514
	Medium	694 (81.26%)	481 (78.98%)	
	High	50 (5.85%)	38 (6.24%)	
Follow‐up information	Visiting time postsurgery (Month)	10.49 ± 7.98	8.86 ± 7.27	0.345

General information and laboratory test results were obtained directly from the HIS. Clinical history, intraoperative findings, and pathological information were manually extracted from semi‐structured electronic reports.

### 2.3 Establishing the two‐tier fusion framework

#### 2.3.1 Stage I: Individual models

##### ML models

Several ML‐based models for cancer prognosis have been developed. Chen et al. recently developed an eXtreme gradient boosting (XGBoost)‐based framework to identify patients with early‐stage pancreatic cancer using clinical data from EHRs.^18^ Wu et al.^19^ established a support vector machine (SVM) model to classify metastatic and non‐metastatic osteosarcoma patients. A risk‐scoring model was developed to quantify the risk by extracting and classifying independent prognostic genes. In this study, five mainstream ML models, including SVM, K‐nearest neighbors (KNN), decision tree (DT), random forest (RF), and extra trees were fine‐tuned and comprehensively evaluated for their performance in predicting the risk of LMs.

##### NLP models

**FIGURE 3:**
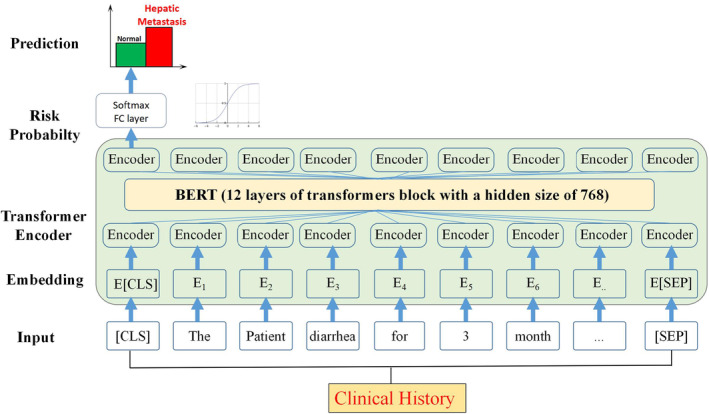
BERT model framework applied in the study. BERT stands for bidirectional encoder representations from transformer. The figure illustrates the flow of information from the input sequence, through the stacked transformer encoder blocks, and finally to the fully connected layer for prediction.

NLP models are pretrained models that have achieved great success, and the bidirectional encoder representations from transformer (BERT) architecture proposed by Google researchers is the most representative. By applying an attention‐based two‐layer transformer architecture, BERT^19^ makes the model parameters fit the text context through unsupervised learning. Central to its design is the [CLS] token, which, influenced by all tokens in the input sequence owing to the self‐attention mechanism of BERT, captures a comprehensive representation of the entire input sequence. This feature is critical for tasks that require the entire context to be understood in our study. In this study, a Chinese BERT model based on Chinese super‐large prediction was adopted and fine‐tuned to evaluate the model's performance in predicting LMs in patients using Chinese‐text medical records. Figure 3 illustrates the framework for fine‐tuning the BERT model used in this study.

#### 2.3.2 Stage II: Fusion models

The feature data from a single modality are not sufficient to assess the patient's condition. For example, laboratory tests provide information about the quantitative changes in tumor markers, but only for a certain period, while free‐text medical records document the long‐term medical experience of patients. Therefore, this study strived to integrate and utilize heterogeneous data by establishing effective fusion frameworks. We used two of the most commonly used fusion schemes to evaluate the effect of different data fusion methods comprehensively.

##### Early fusion (EF) model (BERT‐clinical EF model)

In Stage I of our model, we separately trained ML models on structured clinical data and the BERT model on free‐text clinical notes to maximize the utilization of both clinical and text features, aiming to obtain the best models to fit the predicted LM status. For early fusion (EF), we integrated the vector from the last layer of the BERT model and the features from the best model into a single feature vector. This combined feature vector was then used to train a final XGBoost model for further prediction tasks (Figure 1; Stage II EF). The XGBoost classifier is a powerful ensemble model based on a tree structure and an optimized version of the gradient boosting tree method which incorporates an improved second‐order derivative loss function, regularization term to prevent overfitting, and parallel computing for block storage optimization. The formula for the XGBoost model is shown in Equation 1.

(1) $$L=\sum_{i=1}^{n}l\left(y_{i},,,\hat{y_{i}}\right)+\Omega \left(f\right)$$

where *L* is the loss function, *y* is the actual value, *ŷ* is the predicted value, *l* is the logistic loss function, and *Ω*(*f*) is the regularization term.

##### Late fusion (LF) model (BERT‐clinical LF model)

In the late fusion (LF) model, we use the predictions generated by the models trained in Stage I to reach the final decision (Figure 1; Stage II LF). These predictions, which are derived from the output of the models in Stage I, are then fused using an aggregation function to yield a final result. The aggregation can be achieved using methods such as averaging, majority voting, or weighted voting. The formula for weighted voting is shown in Equation 2.

(2) $$\hat{y}=\frac{\sum \;w_{i}\cdot p_{i}}{\sum \;w_{i}}$$

where ŷ is the final prediction, *w*ᵢ is the weight of each model, and *p*ᵢ is the prediction of each model.

In our LF model, the weights assigned to each model for the weighted voting method were determined based on the performance of the respective models during the training phase. Specifically, the weights were computed as the reciprocal of the error rate observed in the cross‐validation of each model. Hence, models demonstrating lower error rates (indicating higher performance) were assigned greater weights. This method of weight assignment ensures that models with higher performance have a more substantial impact on the final prediction. The detailed equations and explanations are provided in Data S3.

The advantage of the LF approach lies in its ability to integrate independent predictions from multiple models and establish a threshold based on the number of accurately predicted models. Considering the number of models in our study and recent research focusing on LF models, we chose the weighted voting method as the algorithm for LF, offering a more informed and robust final prediction.

### 2.4 Visualization and explanation

SHAP analysis is a method to address model interpretability.^20^ It is based on Shapley values, a game‐theoretic concept developed by economist Lloyd Shapley to determine the importance of individuals by calculating their contributions to cooperation. This method has received much attention in AI interpretability research and has contributed significantly to advancing the clinical applications of models.^21^, ^22^ The Shapley value interpretation is an additive feature attribution method that interprets a model's predicted value as a linear function of a binary variable.

(3a) $$g\left(z^{\prime }\right)=\phi_{0}+\sum_{j=1}^{M}\phi_{j}z_{j}^{\prime }$$

(3b) $$z^{\prime }\in {\left\{0，1\right\}}^{M}$$

(3c) $$\phi_{j}\in R$$

where *g* is the explanatory model (3a), *z* is the coalition vector, *M* is the maximum coalition size (3b), and *ϕ j* ∈ *R* is the feature attribution of feature *j*.

In this study, we employed SHAP analysis to visualize and evaluate the importance of each feature in the EF model and the final decision step to screen the most predictive features. The identified features were used to improve the model's interpretability.

### 2.5 Nomogram modeling

A quantifiable and practical clinical assistance tool is needed to help clinicians identify patients at high risk of developing LMs and implement individualized screening and diagnosis strategies. Therefore, we constructed a nomogram based on the 13 compelling predictive features identified by the SHAP analysis. The nomogram was constructed using the Python system's “rpy” and “rms” packages (Python Software Foundation, version 3.1.1).

## 3 RESULTS

### 3.1 Evaluation method

The performance of each method was evaluated using the ROC curve, along with the accuracy, precision, recall, and F1 scores. Furthermore, true positive (TP) and false positive (FP) are the numbers of correctly and incorrectly predicted positive cases, respectively, while true negative (TN) and false negative (FN) are the numbers of correctly and incorrectly predicted negative cases, respectively. Equations (4a), (4b), (4c), (4d) describe the performance metrics.

(4a) $$\text{Accuracy}=\frac{\mathrm{TP}+\mathrm{TN}}{\mathrm{TP}+\mathrm{TN}+\mathrm{FP}+\mathrm{FN}}$$

(4b) $$\text{Precision}=\frac{\mathrm{TP}}{\mathrm{TP}+\mathrm{FP}}$$

(4c) $$\text{Recall}=\frac{\mathrm{TP}}{\mathrm{TP}+\mathrm{FN}}$$

(4d) F1−score=2precision*recall1precision+1recall

### 3.2 Two‐tier fusion framework

#### 3.2.1 Stage I: Individual models

##### ML models

To explore the potential of predicting the risk of LM using only clinical indicators, five different ML models were first built using structured or semi‐structured clinical data, and the parameters were optimized. Features with significant correlations were excluded using Pearson correlation analysis. None of the 18 clinical features showed linear correlations using Pearson's coefficient (Data S1; Figure S1); hence, they were incorporated into the ML model.

**FIGURE 4:**
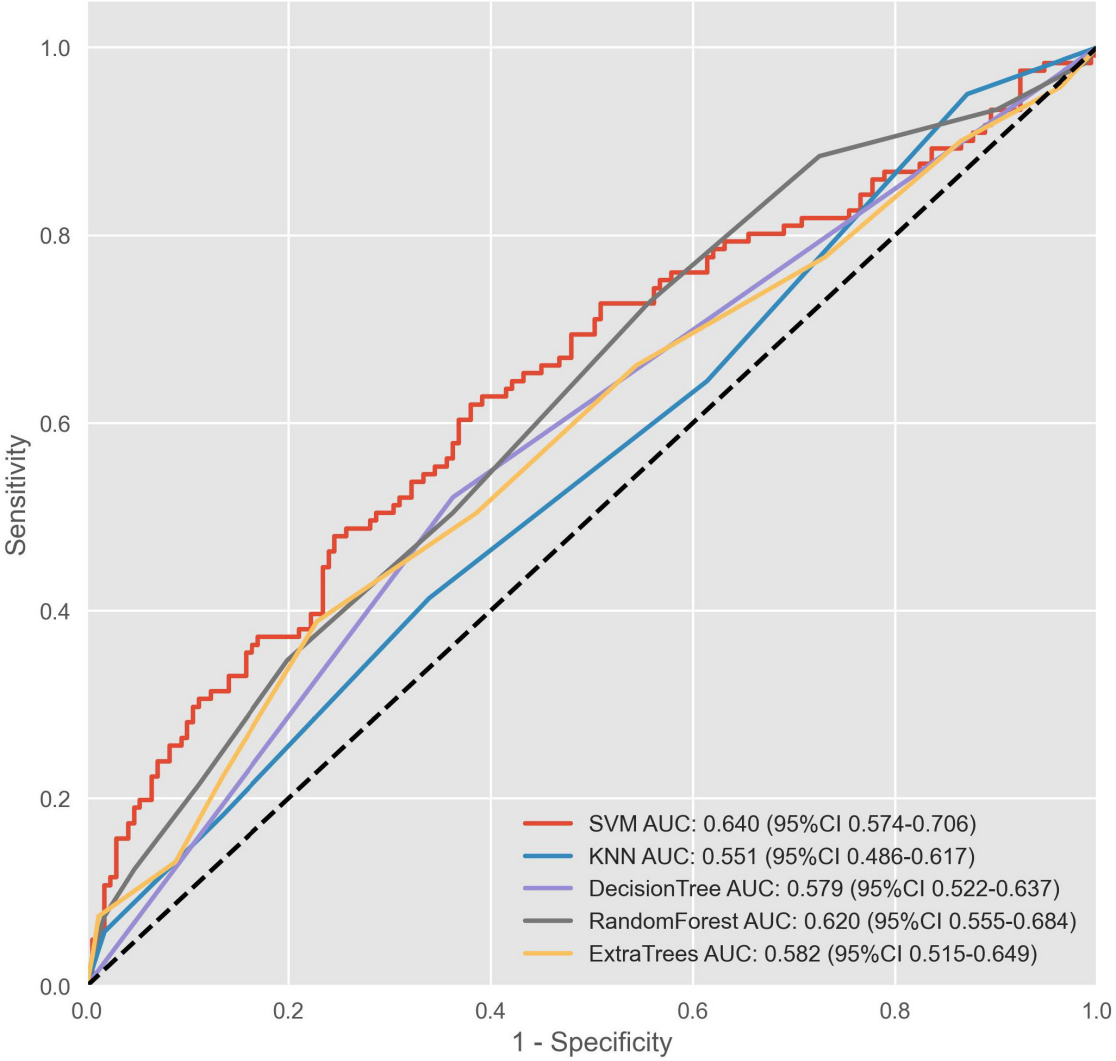
ROC curve and AUC values of machine learning models. AUC, area under the curve; ROC, receiver operating characteristic; SVM, support vector machine; KNN, K‐nearest neighbors.

The ROC curves and AUC values of the five ML algorithm‐building models on the test set are shown in Figure 4, and the accuracy, precision, recall, and F1 values are listed in Table 2. Overall, the performance of each ML algorithm in the validation group was similar and moderate; SVM showed the highest average AUC (0.640) and accuracy (0.640), while the KNN and DT had high recall (0.950) and precision (1.00). However, the F1 values of these two models were lower than their optimal metric (0.230 and 0.685), suggesting a potential deficiency in robustness. Therefore, SVM is considered the preferred optimal ML algorithm and is included in the EF of the second stage.

**TABLE 2:** Comparison of metrics in machine learning models.

Model	Accuracy	Recall	Precision	F1
Support vector machine	**0.640**	0.620	0.620	0.620
K‐nearest neighbors	0.558	**0.950**	0.131	0.230
Decision tree	0.589	0.521	**1.000**	**0.685**
Random forest	0.613	0.727	0.447	0.554
Extra trees	0.613	0.388	0.776	0.518

*Note*: The peak of each index is shown in bold.

##### NLP model

**FIGURE 5:**
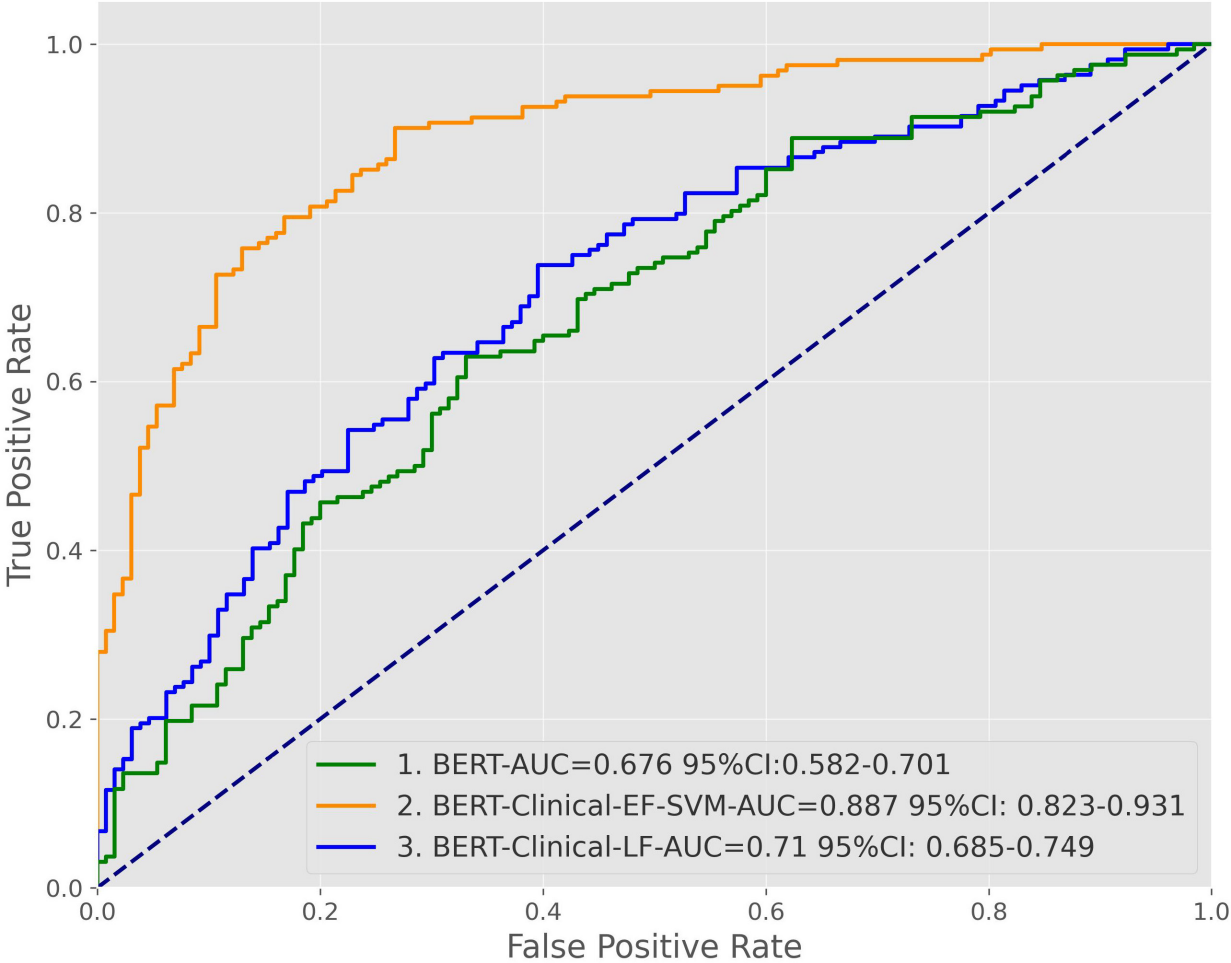
ROC curve and AUC values of the BERT and two fusion models. AUC, area under the curve; BERT, bidirectional encoder representations from transformer; CI, confidence interval; ROC, receiver operating characteristic.

As the NLP model, we used the BERT model with a bidirectional transformer structure, which has received sufficient attention and recognition in medical natural language research. After training, the BERT model obtained a precision of 0.617, recall of 0.613, accuracy of 0.636 (Table 3), and AUC of 0.676 (Figure 5). The BERT model had a more balanced prediction ability for positive and negative samples than the ML model. However, the effect was insignificant compared to the ML model, suggesting that the text features may be valuable for predicting LM but need to be supplemented by other features.

**TABLE 3:** Comparison of metrics in the BERT and two fusion models.

	Accuracy	Precision	Recall	F1
BERT‐fine‐tune	0.636	0.617	0.613	0.624
BERT‐Clinical‐EF‐SVM	**0.808**	**0.803**	**0.805**	**0.808**
BERT‐Clinical‐LF	0.666	0.666	0.645	0.643

*Note*: The peak of each index is shown in bold.

#### 3.2.2 Stage II: Fusion models

In Stage II, we explored two fusion approaches to integrate the ML and NLP models from Stage I. Early fusion concatenated the feature vectors from the ML and NLP models into a single vector to train an XGBoost classifier. Late fusion aggregated the predictions from each model using weighted voting, with weights based on cross‐validation performance. The aim was to fuse the complementary structured clinical and free‐text information to improve predictive ability over individual models.

### 3.3 SHAP analysis

**FIGURE 6:**
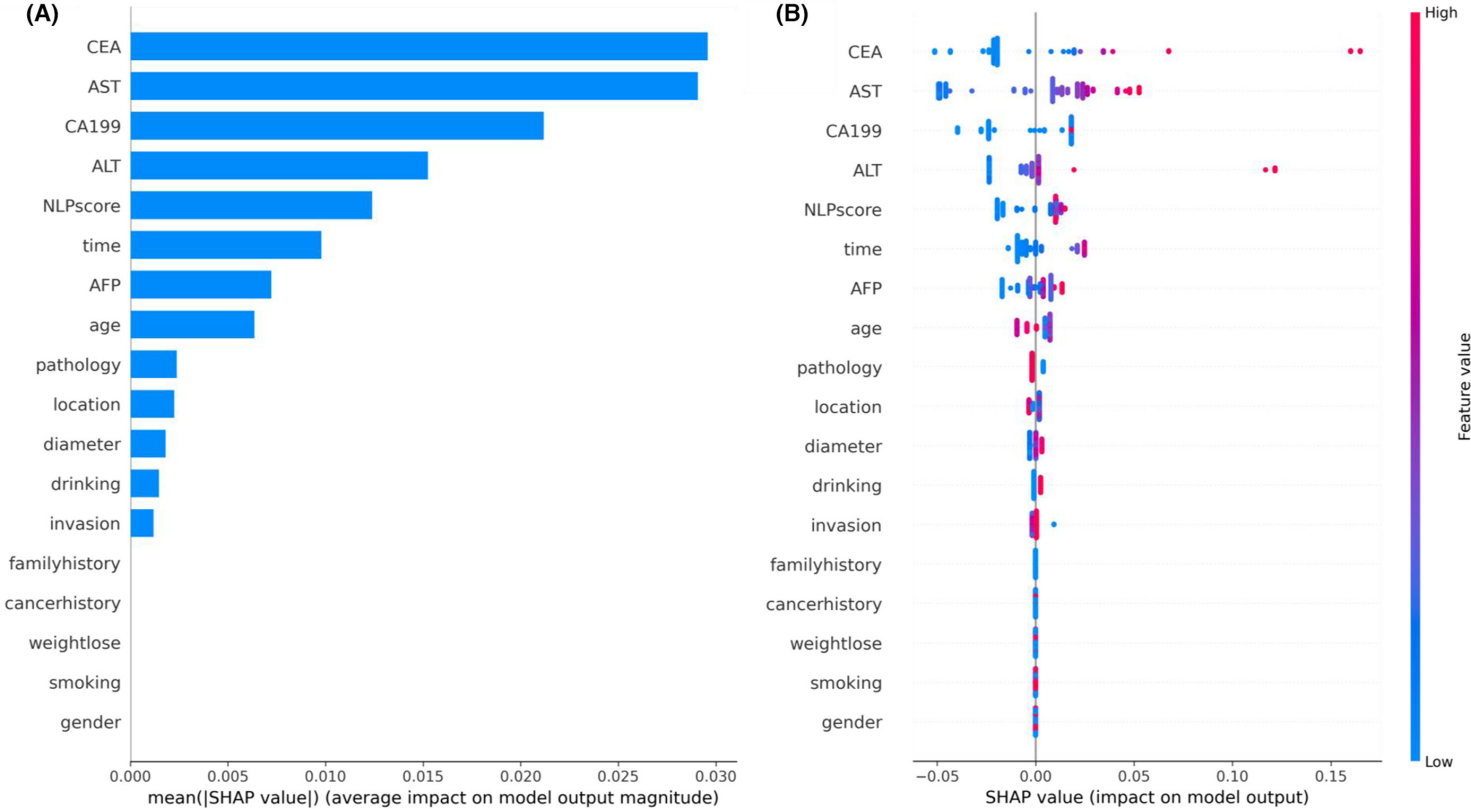
(A) The summary bar plot shows the global importance of each feature in the early fusion model. (B) The summary bee‐swarm plot shows the global importance of each feature and the distribution of effect sizes in the whole test dataset.

Based on the above results, we performed SHAP analysis to evaluate and interpret the impact of different features in the BERT‐clinical‐EF model for predicting CRC liver metastases. As shown in the SHAP summary plot (Figure 6A), four laboratory markers were the strongest predictors of LMs. These included two oncological biomarkers (CA199 and CEA) and two liver enzymatic parameters (ALT and AST), consistent with most clinical studies predicting LMs. It is worth noting that the importance of the “NLP score” is second only to laboratory data, indicating that complex clinical text features provide essential decision‐making information, although this information is not yet fully utilized. In the SHAP summary plot (Figure 6B), all eigenvalues are represented in blue (low) or red (high), and the distance of each point from 0 (SHAP value) represents its contribution (different degrees) to the outcomes, with increasing values favoring the negative (no LM) or positive (LM) classes, respectively.

### 3.4 Nomogram construction

**FIGURE 7:**
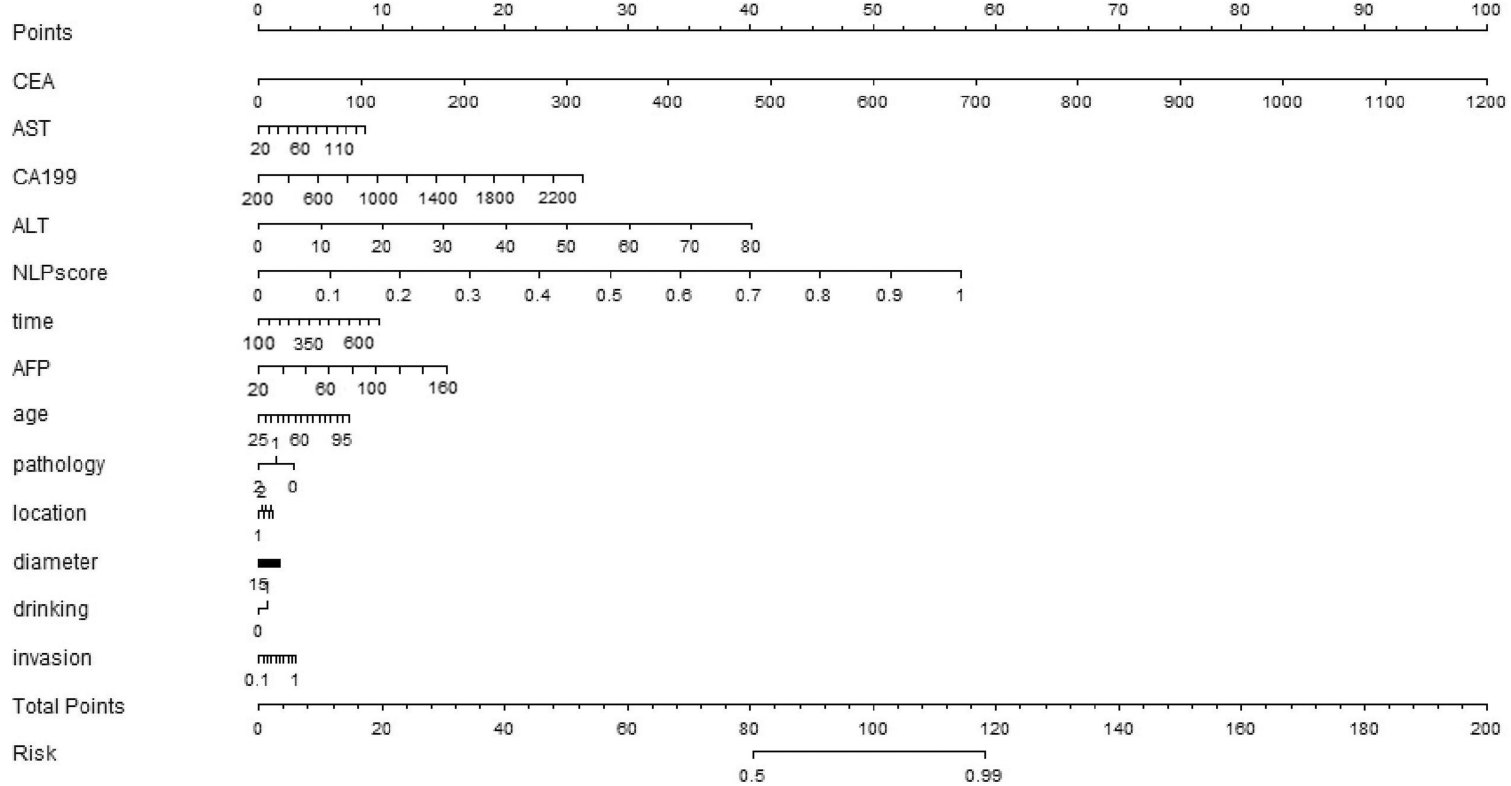
Nomogram of features established by the BERT‐clinical‐early‐fusion model.

Based on the top 13 valid predictors identified by the SHAP analysis, a nomogram was developed to predict the risk of LMs. As shown in the nomogram presented in Figure 7, the effect of each feature on the outcome was consistent with its importance ranking determined by the SHAP analysis.

### 3.5 Nomogram model validation

To validate the predictive performance of the nomogram, an external dataset of 102 cases was collected from the Aerospace Center Hospital. Two physicians, Liu Wenjuan and Lv Han, who have at least 10‐year experience in CRC diagnosis, were blinded to the dataset and participated simultaneously in the validation process.

**FIGURE 8:**
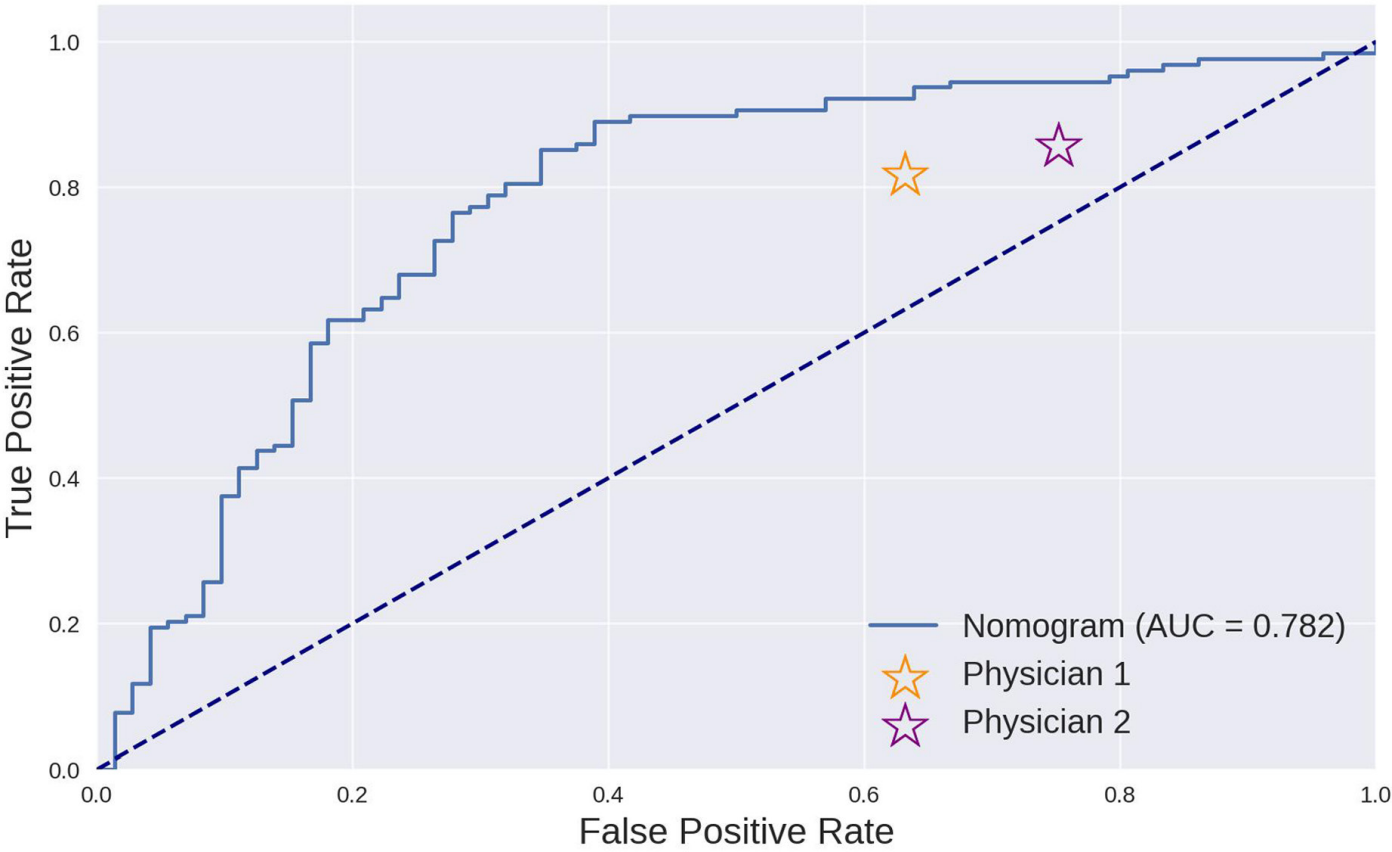
Comparison of the ROC curve of the nomogram and the results of two physicians.

In this external validation, the nomogram demonstrated superior performance compared to the two physicians across key predictive performance metrics, reinforcing its potential utility in predicting the risk of LMs in clinical practice. The ROC curve of the nomogram, presented in Figure 8, yielded an AUC of 0.782, indicating a strong discriminative ability of the model. Compared with the performance of the physicians, represented by two points on the ROC curve, the nomogram achieved a higher TP rate for a given FP rate across a range of threshold probabilities.

Table 4 presents a summary of the key performance metrics for the nomogram and the two physicians. The nomogram consistently demonstrated higher performance across all metrics, underscoring its potential utility in a clinical setting. These results provide evidence supporting the application of the nomogram in clinical decision‐making while also highlighting areas for potential improvement in future iterations of the model.

**TABLE 4:** Comparison of evaluation metrics of the nomogram and two physicians.

	Accuracy	Precision	Recall	F1
Nomogram	**0.760**	**0.763**	**0.906**	**0.829**
Physician 1	0.658	0.697	0.820	0.754
Physician 2	0.640	0.670	0.860	0.753

*Note*: The peak of each index is shown in bold.

## 4 DISCUSSION

Considering the escalating global incidence of CRC, there is an urgent need for tools capable of quantifying the risk of disease progression, ultimately enhancing overall patient outcomes. A significant clinical challenge lies in accurately determining the risk of CRC‐related LMs and conducting timely imaging screening.^21^ Numerous studies employing ML and AI technology^22^ have contributed to the improved prognosis of CRC patients with remarkable results. However, the majority of these studies rely on costly high‐throughput sequencing genetic data or high‐quality imaging or pathology data.^23^ By contrast, medical free texts,^24^ representing the most prevalent and effective data indicative of patient disease progression, have been largely overlooked. With advances in NLP technologies such as BERT,^25^ computers are increasingly adept at understanding human language, and medical free text is poised to become another major branch of omics research.

In this pioneering study, we introduced a fusion modeling approach that combines textual and clinical data to predict the risk of LMs in patients. Notably, to the best of our knowledge, this is the first study to merge NLP and classical ML prediction methods in the oncology domain. In the first stage, we employed five classic ML models to predict LMs but observed suboptimal results, suggesting that laboratory tests alone were insufficient for the prediction.^26^ In the second stage, we experimented with two levels of data fusion between the trained NLP and ML models. We found that the EF of models proved more effective than LF. This could be attributed to the ability of EF to preserve and incorporate the information from textual data into the decision model at an earlier stage, allowing for a more integrated and comprehensive representation of the data. By contrast, LF, which combines the predictions from individual models at a later stage, may not fully leverage the interactions between the different types of data.

A critical barrier to the clinical application of deep learning is the “black box” nature of AI models.^27^ To address this issue, we assessed feature importance in model decision‐making using the state‐of‐the‐art SHAP algorithm.^28^ In the top‐performing EF models, tumor biomarkers and liver enzymes emerged as the most crucial factors for decision‐making compared with other indicators, aligning with previous CRC clinical study conclusions.^29^, ^30^, ^31^ Furthermore, these findings are consistent with existing clinical evidence^32^ and perspectives^33^ on CRC, underscoring the value of these indicators. Notably, both the SHAP interpretation map and the nomogram map revealed that the clinical text features (NLP score) processed by NLP technology played a relatively significant role in decision‐making. By contrast, medical free texts,^34^ the most common and effective data reflecting patient disease progression, have been underappreciated. With breakthroughs in NLP technologies such as BERT, computers will further improve their ability to comprehend human language, leading to medical free text becoming another vital branch of omics research.^35^, ^36^

This study has several limitations that warrant further investigation. Most importantly, due to technical constraints and hardware resources, we used a fine‐tuned version of the BERT model rather than more advanced methods, such as domain pretraining. Consequently, the model may have limitations in understanding free‐text medical records. Additionally, the data scale in this study was relatively small compared to similar studies, which may introduce biases that could affect the robustness of the model. We also acknowledge that while the SHAP algorithm provides some level of interpretability, it does not fully explain the “black box” nature of our model, highlighting the need for caution in interpreting the conclusions drawn from the SHAP analysis in our study. Finally, we believe the model architecture still has room for improvement, such as adopting the BioBERT architecture proposed by Lee et al.^37^ or the Siamese network architecture suggested by Bajaj et al.^38^ Exploring data fusion methods will enable the development of efficient prognostic models for multimodal data to improve human health in the oncology field.

## 5 CONCLUSIONS

We developed a fusion framework based on NLP and clinical data to predict the risk of postoperative metastasis in CRC patients. Our EF model outperformed standalone ML‐ and NLP‐based models. In addition, we utilized the SHAP method to verify the interpretability of clinical and textual data and demonstrated their critical role in the final decision‐making. We also built a quantitative nomogram map for clinical practice based on our model. We believe our findings will promote the application of NLP and data fusion techniques in oncology to improve clinical decision‐making and overall patient outcomes.

## AUTHOR CONTRIBUTIONS

**Jia Li:** Conceptualization (lead); formal analysis (lead); software (lead); visualization (lead); writing – original draft (lead). **Xinghao Wang:** Data curation (equal); formal analysis (supporting); methodology (equal); writing – original draft (supporting). **Linkun Cai:** Data curation (equal); formal analysis (equal); supervision (equal); validation (equal). **Jing Sun:** Conceptualization (equal); investigation (equal); methodology (lead). **Zhenghan Yang:** Funding acquisition (equal); resources (equal); supervision (equal). **Wenjuan Liu:** Data curation (equal); funding acquisition (lead); investigation (lead); writing – review and editing (lead). **Wang Zhenchang:** Funding acquisition (lead); investigation (lead); supervision (lead); writing – review and editing (lead). **Han Lv:** Conceptualization (lead); data curation (lead); funding acquisition (lead); investigation (lead).

## FUNDING INFORMATION

This research was funded by Grant 61931013 (Wang Zhenchang), 62171297 (Lv Han), and 82202258 (Liu Wenjuan) from the National Natural Science Foundation of China, Beijing Hospitals Authority Clinical Medicine Development of Special Funding Support No: ZYLX202101, Beijing Municipal Science and Technology Commission [Grant Number Z201100005620009], and Beijing Postdoctoral Research Foundation [2022‐ZZ‐001].

## CONFLICT OF INTEREST STATEMENT

The authors declare no conflict of interest

## ETHICS STATEMENT

The study was conducted according to the Declaration of Helsinki. It was approved by the Beijing Friendship Hospital Ethics Committee, Capital Medical University (Research Application System number 2021‐P2‐144‐01) and by the Ethical Review of Biomedical Research Involving People, the Ministry of Public Health of China.

## INFORMED CONSENT STATEMENT

Not applicable.

## Supporting information


Data S1

**Data S2**: Click here for additional data file.

Data S3:

## Data Availability

The datasets generated and analyzed during the current study are not publicly available because of the institution's policies involved in human genetics resources, but a limited sample is available from the corresponding author upon reasonable request.
