# Supplementary material for: An interpretable deep learning framework for predicting liver metastases in postoperative colorectal cancer patients using natural language processing and clinical data integration
Source: Cancer Med. 2023 Sep 11;12(18):19337–51. doi: 10.1002/cam4.6523 (PMC10557887; doi:10.1002/cam4.6523)

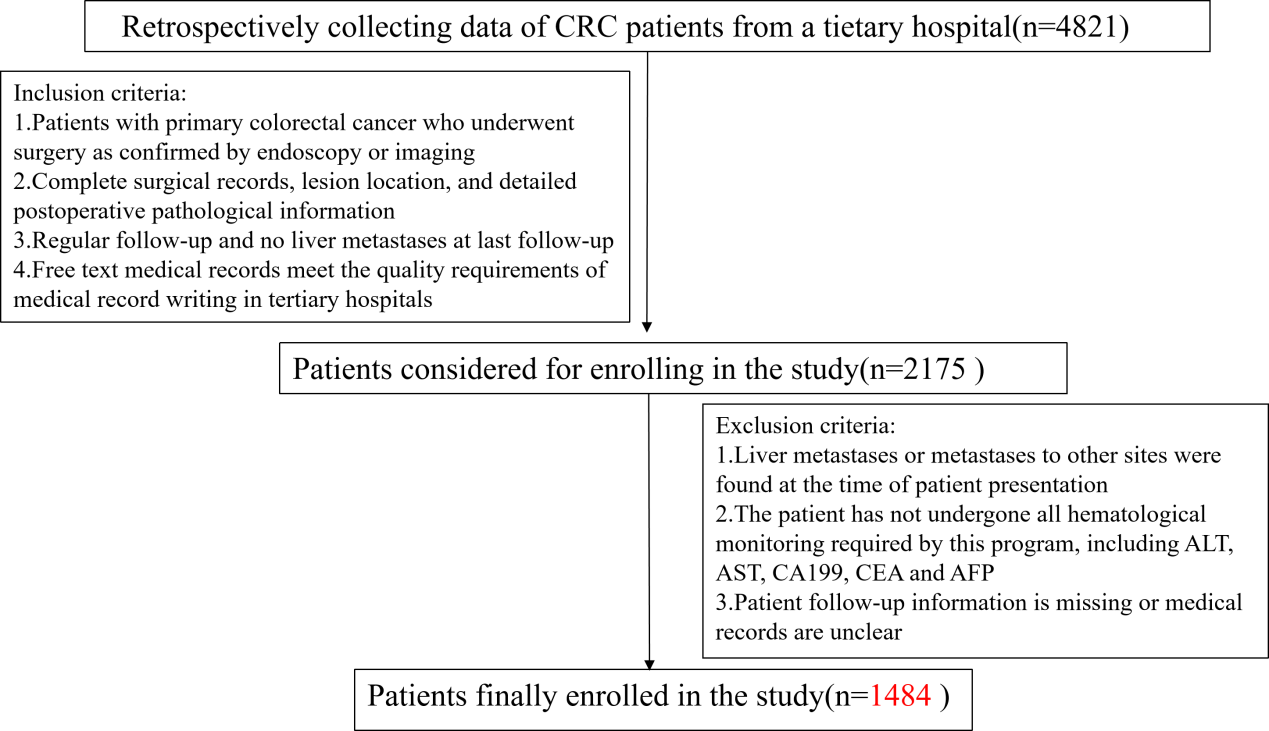


**Table S1 The patient enrollment roadmap for this study**

**Figure S1. The Pearson's coefficient analysis in 18 clinical structured data, coefficient ≥0.9 is refered as a threshold for linear relationship, linear relationship is not observed between 18 factors.**


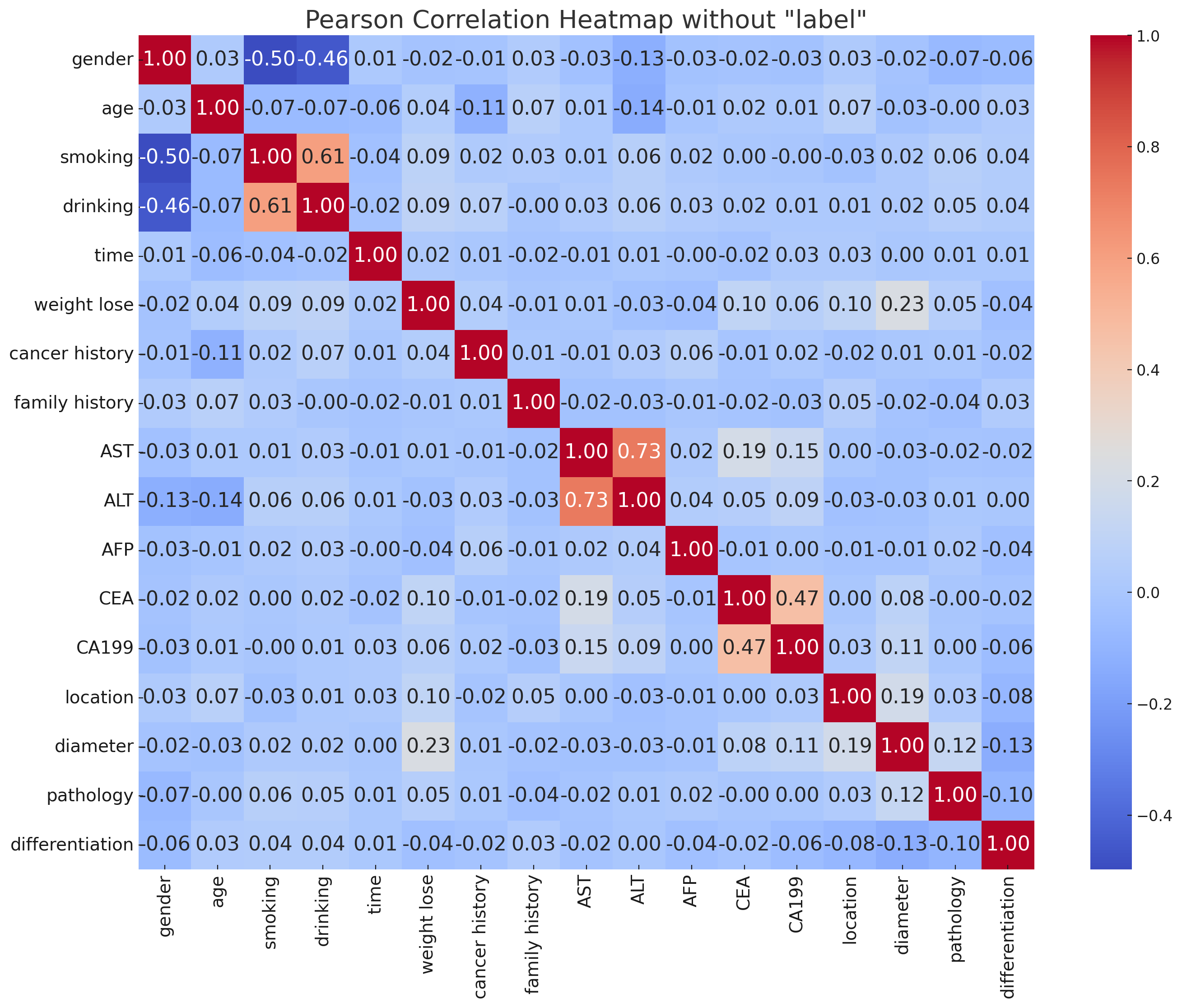

Supplement: Supplementary file 1 — Data S1 [file CAM4-12-19337-s002.docx]
