# Supplementary material for: An interpretable deep learning framework for predicting liver metastases in postoperative colorectal cancer patients using natural language processing and clinical data integration
Source: Cancer Med. 2023 Sep 11;12(18):19337–51. doi: 10.1002/cam4.6523 (PMC10557887; doi:10.1002/cam4.6523)
Supplement: Supplementary file 3 — Data S3: [file CAM4-12-19337-s003.docx]

### Supplementary Material 3

The weights assigned to each model in the ensemble were derived based on their cross-validation performance using the following equations:

Equation S1

$$w_{i}=\frac{1}{{\{error\}}_{i}}$$

where $w_{i}$ is the weight for $\mathrm{model}_{i}$ and ${error}_{i}$ is the error rate of $\mathrm{model}_{i}$observed in cross-validation. Hence, models demonstrating lower error rates (indicating higher performance) were assigned greater weights. This method of weight assignment ensures that models with higher performance have a more substantial impact on the final prediction.

Equation S2

$error=$*k*$\times(1-$*Average* $F1 Score)+$*β*$\times Standard Deviation$

In Equation S2, the error for each model was calculated by average F1 and standard deviation across the 5 folds of cross-validation(see Table S2). The coefficients α and β were optimized through grid search (https://scikit-learn.org/stable/modules/grid_search.html) to identify the combination that maximizes the ensemble's overall performance on the validation set. The optimal values were determined to be α=4 and β=8(Equation S3).

.Equation S3

$error=4\times(1-$*Average* $F1 Score)+8\times Standard Deviation$

As shown in Table S2, the F1 score, standard deviation, and error rate were computed for each model using their 5-fold cross-validation results. These values were then applied in Equations S1-S3 to derive the ensemble weight for each model. Therefore, models demonstrating lower error rates (indicating higher and more stable performance) were assigned greater weights, ensuring they have a larger impact on the final ensemble prediction.

Table S2 final weights for models in Late Fusion

| Model | F1 value of Cross-validation on training set | | | | | | | Error | Weights |
| --- | --- | --- | --- | --- | --- | --- | --- | --- | --- |
|  | CV 1 | CV 2 | CV 3 | CV 4 | CV 5 | Average | Standard Deviation |  |  |
| BERT | 0.813 | 0.636 | 0.711 | 0.874 | 0.826 | 0.772 | 0.086 | 1.603 | 0.624 |
| SVM | 0.635 | 0.900 | 0.749 | 0.755 | 0.826 | 0.773 | 0.088 | 1.613 | 0.620 |
| KNN | 0.057 | 0.928 | 0.021 | 0.800 | 0.079 | 0.377 | 0.400 | 5.692 | 0.176 |
| Decision tree | 0.788 | 0.999 | 0.586 | 0.739 | 0.373 | 0.701 | 0.216 | 2.923 | 0.342 |
| Random Forest | 0.581 | 0.750 | 0.761 | 0.677 | 0.836 | 0.721 | 0.086 | 1.805 | 0.554 |
| Extra trees | 0.556 | 0.670 | 0.652 | 0.784 | 0.779 | 0.688 | 0.085 | 1.931 | 0.518 |
